# Bone marrow biopsy and aspiration: a departmental financial comparison in a rural hospital

**DOI:** 10.1186/s13104-021-05491-1

**Published:** 2021-03-04

**Authors:** Nancy W. Bethuel, Eric Bravin, Nicole Krupa, Paul Jenkins, Melissa Scribani, Anne Gadomski

**Affiliations:** Bassett Medical Center, One Atwell Road, Cooperstown, NY 13326 USA

**Keywords:** Bone marrow biopsy, Bone marrow aspirate, Rural health, Hematology oncology, Interventional radiology

## Abstract

**Objective:**

The purpose of this study was to compare the charges and payments associated with bone marrow aspiration and biopsies performed by hematology/oncology specialists versus interventional radiology specialists at Bassett Medical Center located in a rural area of New York State. Charges pertained to what the hospital charged for the procedure and payment refers to the reimbursement the hospital received. Our secondary objectives were to compare specimen quality by procedure and to determine whether body mass index was associated with which specialist performed the procedure.

**Results:**

The median charge was significantly higher in the interventional radiology group ($5254 USD) compared to the hematology/oncology group ($413 USD), *p* < 0.0001. Median payments were also higher in the interventional radiology ($1392 USD) compared to the hematology/oncology group ($1109 USD), *p* < 0.0001. Adequacy of the samples obtained by either profession was similar. Disease process was not associated with adequacy of the sample. There was no statistically significant difference in the proportion of males and females in the respective groups or in the mean age. However, the patients’ in the interventional radiology group had a significantly higher mean BMI (34.3) compared to those in the hematology/oncology group (28.6), *p* = 0.0014.

## Introduction

Bone marrow aspiration and biopsies (BMAB) procedures are carried out for cytological assessment of various hematological diseases [1]. Highly specialized testing, such as cytogenetic, immunophenotypic, and molecular analyses, can be performed on these specimens; this information has become critically important in establishing certain diagnoses, especially leukemia and lymphomas.

The procedure is carried out by a trained clinician so that adequate tissue is obtained for evaluation [2]. Biopsies are usually performed on the posterior iliac crest. While patient comfort and safety are important, health care related cost is also important especially in rural settings and/or low resource settings.

Over the last few years, we have noted an increasing trend in the number of BMAB procedures performed by interventional radiology (IR) specialists [3] compared to those performed in office by hematology/oncology (HO) specialists. We hypothesized that this cost would be significantly higher in the IR department with no significant difference in procedure success or sample adequacy.

The purpose of this study was to analyze the costs associated with BMAB performed by HO compared to IR in our rural health network between April 2017 and March 2019. A secondary objective was to describe the differences in patient characteristics for those whose BMAB was done by IR compared to those that were done by HO.

## Main text

### Methods

All pathology reports for BMAB performed between April 2017 and March 2019 by HO and IR at our institution were retrospectively reviewed by the authors. Because costs cannot be measured directly, we compared two proxies for the health-related costs: 1) charges pertain to how much the patients were charged for the procedure and 2) payments refers to the reimbursement the hospital received.

The study population included inpatients and outpatients. Each procedure was reviewed for age, sex, BMI, diagnosis and adequacy of the sample. Charges associated with each procedure encounter were compiled by our finance department. We merged biopsy data with finance data based on hospital identification number (ID). Medical record numbers (MRNs) with more than one hospital ID (more than one biopsy) were included. One set of duplicate hospital IDs was deleted because there were two records of biopsies on the same contact date; the finance department confirmed that this was a duplicate report. Since we had no way to know which biopsy to assign that cost to, both were deleted. One was in the HO group and one in the IR group.

Due to large right skew in the distribution of charges and payments, we presented medians and interquartile ranges. For MRNS with multiple biopsies, age, BMI and cost data were averaged and weighted by the number of biopsies per MRN. Comparisons of mean age and mean BMI between the HO and IR groups were carried out using the student’s t-test. The chi-square test was used to compare the percentage of male/female patients between HO and IR groups.

The non-parametric Wilcoxon Rank Sum test was used to compare median charges and payments between HO and IR groups. P-values of less than 0.05 were considered statistically significant. The number of procedures performed by HO and IR groups, respectively, were examined for change over the study period using linear regression analysis. A significant positive slope would indicate an increasing number of procedures, while a significant negative slope would indicate a decreasing number of procedures over time.

This study was deemed exempt from continuing review by the Mary Imogene Bassett Hospital Institutional Review Board.

## Results

There was a total of 270 procedures performed during the study period; 179 and 91 performed by HO and IR respectively. No immediate complications were reported in either group. Five patients in both groups required repeat procedures to obtain an adequate bone marrow sample. There was no difference in the adequacy of the sample obtained by either method, or no difference in need for repeat procedure. Disease process was not a factor in whether the sample was adequate.

**Fig. 1:**
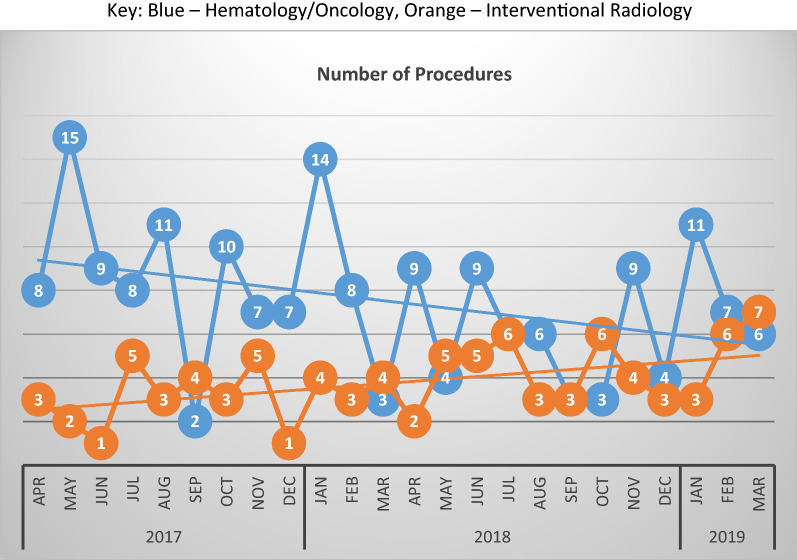
Number of procedures. Key: Blue—Hematology/Oncology, Orange—Interventional Radiology

The number of biopsies performed by IR increased significantly over the study period (*p* = 0.023; Fig. 1). In contrast, the number of biopsies done by HO showed a general, although non-significant (*p* = 0.09), downward trend (Fig. 1).

There was a higher proportion of males in the HO group (Table 1). However, this difference was not statistically significant. Mean BMI was significantly higher among the IR biopsies; 34.3 compared to 28.6 kg/m^2^ in the HO group (Table 1).

**Table 1:** Patient characteristics

	HO	IR	*p *value
Mean Age	66.3	65.0	0.5127
% Males	55.9	46.2	0.1309
Mean BMI	28.6	34.3	< 0.0001
Diagnostic Rate	97.8%	94.5%	N/a
Median Charges (Interquartile Range)	$413 ($192–$2050)	$5254 ($4328–$6227)	< 0.0001
Median Payments (Interquartile Range)	$1109 ($119–$1408)	$1392 ($1122–$3695)	< 0.0001

Median charges were also significantly higher in the IR group (Table 1), $5254 USD (interquartile range: ($4328–$6227) compared to $413 USD (interquartile range: $192–$2050) in the HO group. Payments were higher for the IR group, with median payments of $1392 USD compared to $1109 USD for the HO group (Table 1). The higher charges for procedures by the IR group were likely attributable to operating and recovery room costs.

## Discussion

We compared the health care related costs of a relatively common procedure when performed by HO versus IR. Although sample adequacy was the same, median charges and payments were significantly higher when the procedure was performed by IR specialists compared to in office procedures performed by HO specialists.

Our rural community hospital has seen a steady increase in IR procedures which has raised concerns about the costs. We found that over the last two years, more BMAB are being performed by the IR department with no difference in quality of the specimens. A similar trend has been seen across other parts of the United States [1].

While the World Health Organization recommends a bone marrow length of >  = 15 mm and while operator experience can affect specimen quality [2], we were not able to assess these factors as very few pathology reports commented on the length of the biopsies. The most common reason to repeat a BMAB was an acellular or suboptimal core and dilute or apiculate aspirate. However, in suboptimal samples where the diagnosis was still made, the procedure was not repeated.

Previous studies have reported higher rates of successful and safe BMAB by CT guided biopsy in patients with higher BMIs [3]. This could explain why the IR group of patients have a statistically significant higher mean BMI (Table 1). However, we could not determine by chart review whether our HO specialists systematically referred patients with higher BMIs to IR. When we presented our results to the HO department, we learned that body habitus makes it difficult to palpate physical landmarks without imaging guidance [3]. A potential alternative to an IR procedure would be, ultrasound guided BMAB that has been shown to be just as effective [4], but less costly than an IR procedure. Further research is needed to demonstrate whether ultrasound guided BMAB is as effective as IR in yielding an adequate sample while incurring less cost. Of note, previous studies have not found difference in diagnostic rate of BMAB performed by IR versus HO in non-obese patients [4].

The median cost for IR performed BMAB was $1392USD per BMAB compared to $1109USD per BMAB performed by HO, yielding an average excess cost of approximately $283 USD per BMAB performed by IR. Given similar sample adequacy at a substantially and significantly increased cost, our findings do not support universal performance of IR performed BMAB. CT-guided biopsy also exposes patients to radiation. Having said that, IR performed BMAB could be reserved for patients with body habitus that limits the clinician’s ability to palpate important anatomic landmarks [1]. Alternatively, ultrasound guided BMAB could be used for these patient groups [5], however a comparison of IR guided BMAB and ultrasound guided BMAB is needed.

These findings imply that the cost-effectiveness for care of patients with hematologic disorders could be improved. Although sample adequacy was comparable, median charges and payments were significantly higher when the BMAB procedure was performed by an IR specialist compared to in office procedures performed by HO specialists. Reasons for patient referral to IR may include elevated BMI. Whether ultrasound guided BMAB can be used as an alternative to the more costly IR procedure is unknown but should be investigated. Further research is also needed regarding a potential cut-off BMI at which patients should be referred to IR.

## Limitations

The study was performed at a single institution in a rural area, thus limiting its generalizability to urban settings and different patient populations. Given the increasing prevalence of obesity across the United States [6], there will likely be an increasing trend of CT-guided biopsy with IR. This being a retrospective study itself introduces a bias potentially related to the quality of data collected because these data were not planned ahead of time and standardized. Lastly there may be an imbalance of unmeasured factors between the two groups that influenced the type of BMAB procedure they received. These limitations could be resolved in the careful design of prospective studies in the future.

## Data Availability

The data that support the findings of this study are available on request from the corresponding author, Nancy Bethuel. The data are not publicly available due to their containing information that could compromise the privacy of the research participants.

## Abbreviations

BMAB: Bone marrow aspiration and biopsies
IR: Interventional radiology
HO: Hematology/oncology
ID: Hospital identification number
MRN: Medical record numbers

## References

[1] Manchec B, Limback J, Liu B, Pepe JW, Burt J, Contreras F, Ward TJ (2020). Safety and technical success of CT-guided bone marrow biopsy in obese patients. J Clin Interv Radiol ISVIR.

[2] Marinelli LM, Fang H, Howard MT, Hanson CA, Haack JJ, Eick EA, Allen RJ, Ruffridge DE, Byrne CM, King RL (2018). Bone marrow biopsy operator experience and impact on aspirate, biopsy, and ancillary testing quality. Mayo Clin Proc Innov Qual Outcomes.

[3] Ahmed O, Wadhwa V, Patel MV, Patel K, Lionberg A, Klejch W, Lizardo A, Ginsburg M (2020). Increasing volume of bone marrow biopsies by radiology providers: evaluation of trends by physician specialty and practice setting. J Am Coll Radiol.

[4] McLaughlin S, Mead L, Clark T, Trerotola S, Dagli M. 04: 03 PM Abstract No. 380 Development of evidence-based guidelines for bone marrow biopsy requests in IR: a retrospective comparison of the outcomes of 2800 biopsies done at the bedside versus under image guidance. J Vasc Interv Radiol 2019; 30(3):S167–8.

[5] Raza S, Raza F, Rezaie N, Quiambao J. 04: 12 PM Abstract No. 381 Using ultrasound guidance for bone marrow biopsy in an outpatient office setting. J Vasc Interv Radiol. 2019; 30(3):S168.

[6] Lee M (2020). Obesity among US rural adults: Assessing selection and causation with prospective cohort data. Health Place.

